# ‘What is ‘wellbeing’ to doctors in training, and how do we improve it? Results of a quantitative survey and thematic analysis of internal medicine trainees

**DOI:** 10.1016/j.fhj.2024.100208

**Published:** 2024-11-01

**Authors:** Alexander Emery, Jesal Acharya, Oliver Fox, Emma Rowlandson

**Affiliations:** a Chelsea and Westminster Hospital NHS Foundation Trust, West Middlesex University Hospital, Twickenham Rd, Isleworth TW7 6AF, United Kingdom; b King's College Hospital NHS Foundation Trust, King's College Hospital, Denmark Hill, London, SE5 9RS, United Kingdom; c Royal Free London NHS Foundation Trust, Barnet Hospital, Wellhouse Lane, Barnet, EN5 3DJ, United Kingdom

**Keywords:** Wellbeing, Education, Workforce, Junior doctors, Internal medicine training

## Abstract

**Introduction:**

The wellbeing of medical trainees has come into sharp focus in recent years, particularly since the COVID-19 pandemic. Workforce pressures are well documented, as is the need to retain staff. There are a number of initiatives to try and improve the wellbeing of staff, including the introduction of wellbeing and education dellows for stage 1 internal medicine trainees in some regions. We recognise that there is often a gap between what is offered and what is wanted or accessed by trainees.

**Objective:**

This work aims to identify what ‘wellbeing’ means to this group of staff, and to provide evidence for interventions that they would find useful.

**Methods:**

We distributed a survey to the target group of staff in London, followed by a series of structured interviews and focus groups. We used a practical thematic analysis to explore the determining factors of trainee wellbeing.

**Results:**

156 trainees responded to the survey, and 15 participated in the interviews and focus groups. Seven themes were identified: culture, helplessness and distrust, individual circumstances, physiological needs/safety, rotas and work patterns, stress at work, and value.

**Conclusion:**

This study identifies several key factors related to medical trainee wellbeing. To our knowledge, it is the first looking specifically at stage 1 internal medicine trainees in the UK.

## Highlight

• Healthcare professionals are at a high risk of burnout, and the wellbeing of medical trainees has come into sharp focus in recent years.
• Interventions to improve the wellbeing of medical trainees are welcome, but there are gaps the research around where these interventions should be targeted.
• Internal medicine trainees are a group of doctors 3–5 years post-graduation in the UK, and have been found to be at increased risk of burnout compared to their peers.
• Themes related to poor wellbeing in this group of staff were culture, helplessness and distrust, individual circumstances, physiological needs/ safety, rotas and work patterns, stress at work, and value.
• Our work proposes interventions based on these themes#.

## Introduction

Wellbeing as a concept has been around for decades, but research into this has accelerated rapidly over the last few years. Programs aiming to improve the wellbeing of individuals both at work^1^^,^^2^ and in wider society^3^ are commonplace and attempts to measure and introduce this to healthcare settings are becoming more prevalent.^4^, ^5^, ^6^, ^7^, ^8^ However, there is no standardised definition of wellbeing, an issue that has been recognised for some time.^5^^,^^9^^,^^10^ The World Health Organization (WHO) in its founding constitution linked wellbeing with health when it defined health as ‘A state of complete physical, social and mental well-being, and not merely the absence of disease or infirmity.’^11^ More recently it has attempted to define wellbeing as its own individual concept and agreed on the Geneva Charter for Wellbeing in 2021.^12^ Here, wellbeing was defined as ‘A positive state experienced by individuals and societies. Like health, it is a resource for daily life and is determined by social, economic, and environmental conditions.’^13^

Given the lack of a universally agreed definition, it is unsurprising that there are several ways of attempting to measure wellbeing. The methods employed to measure wellbeing can be qualitative or (quasi-) quantitative.^3^ Other organisations, while not necessarily conducting primary research, have collated bodies of evidence highlighting the scale of the problem. These invariably use proxy measurements of wellbeing such as mental health problems including suicide rates,^14^ alcohol and drug use, sick days from work, and other work-related stressors.^15^ A widely used resource to track UK medical staff wellbeing is the General Medical Council (GMC) national training survey (NTS) .^16^ This mandatory survey is completed by over 70,000 doctors in training each year and provides a score for proxy measurements of wellbeing such as ‘Overall satisfaction’. The NTS also has an optional section on burnout completed by over 40,000 trainees, which takes seven questions from the Copenhagen Burnout Inventory^17^ to create an overall burnout score. In 2023, this showed that over 75% of stage 1 internal medicine trainees (IMTs) scored high or moderate risk, compared with 65% of their peers in other specialty training programmes.^16^ This study aimed to explore the factors influencing the wellbeing of this group of trainees; however, we believe that the findings are applicable to multiple staff groups.

**Fig. 1:**
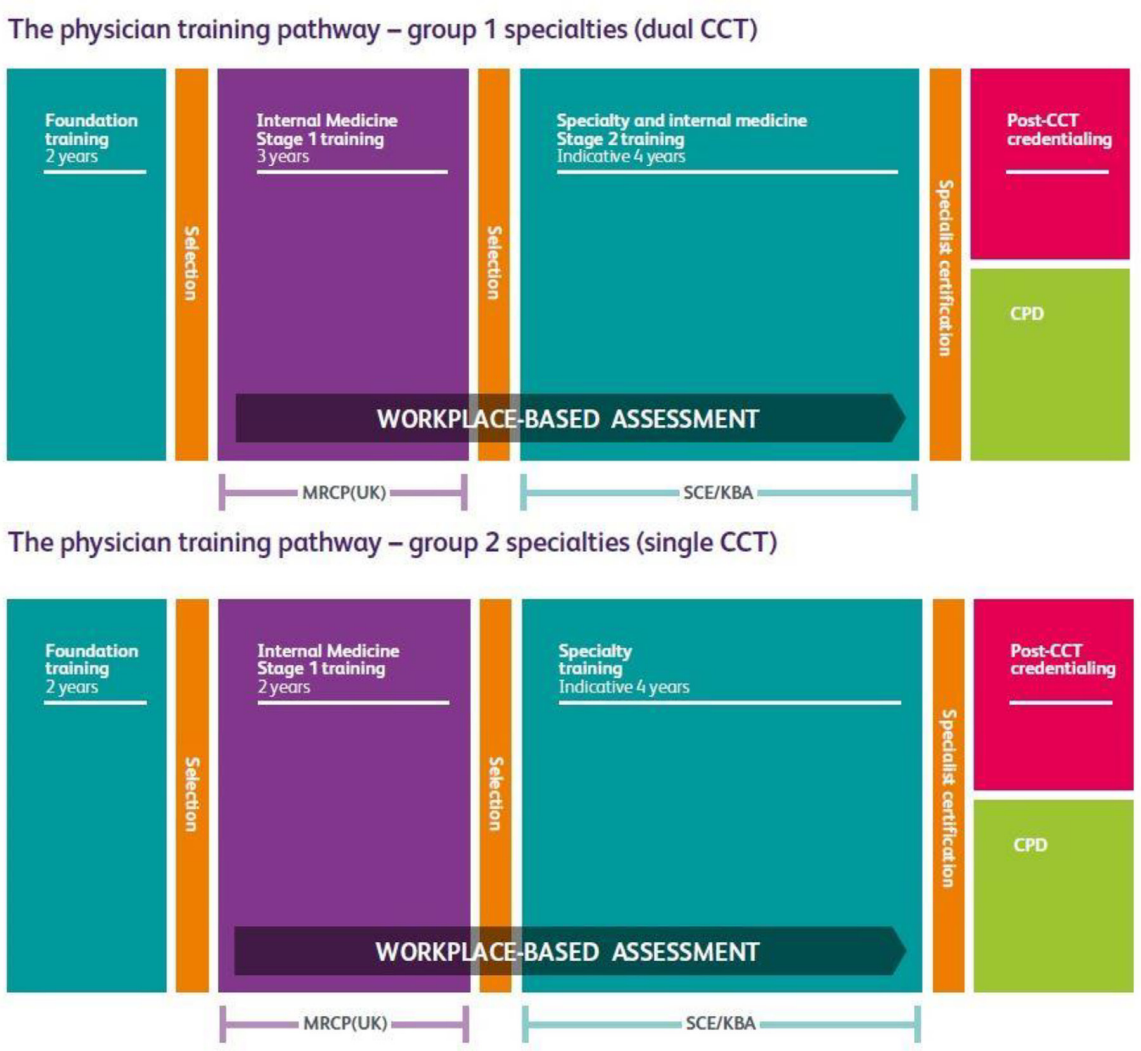
Physician Training Pathways in the UK (reprinted with permission from https://www.thefederation.uk/training/training-certification/training-pathways).

While the above approaches give valuable quantitative data, they do not provide significant insight into trainees’ individual perceptions of the underlying determining factors of their wellbeing.^16^ Other studies have looked into this^18^^,^^19^ but none, to our knowledge, have looked specifically at IMTs. In the UK, stage 1 internal medicine training is a 2- or 3-year training programme that bridges the gap between foundation training (taken immediately after medical school graduation) and specialty training in a medical specialty, as shown in Fig. 1.

Our work surveyed all internal medicine trainees (IMTs) in London and gave the opportunity for individual responses to better understand the causative factors, followed up with more detailed interviews and focus groups to explore the complexity of the issues.

## Methods

### Study design

**Fig. 2:**
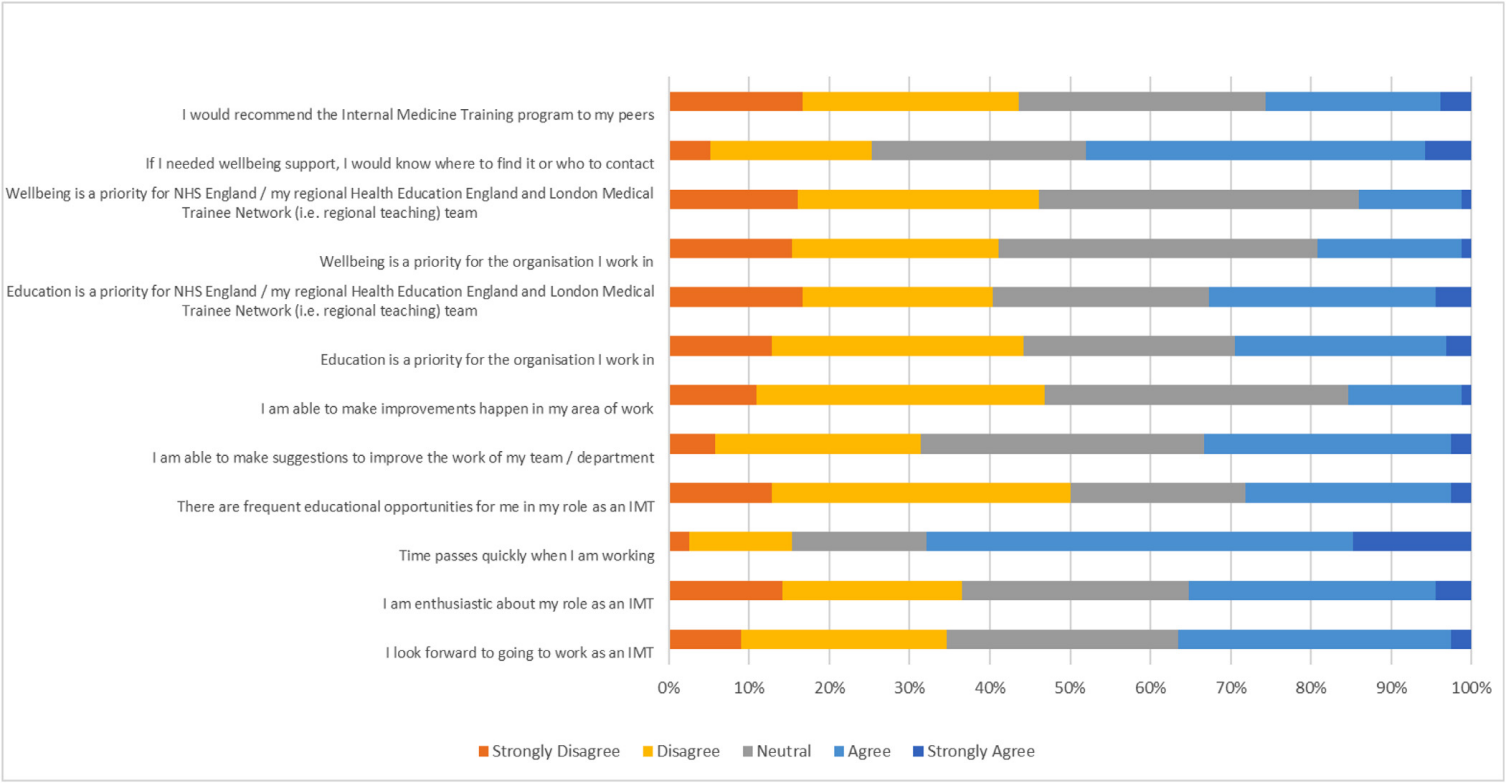
Likert-style responses to all survey question.

Quantitative data were gathered from a regional survey of IMTs, with respondents asked to rate their agreement with statements on a Likert-type scale. These were based on a selection of GMC NTS questions,^16^ but were asked specifically in relation to the respondent's role as an IMT, eg ‘I am enthusiastic about my role as an IMT’ rather than ‘… as a doctor’ (Fig. 2) .

We used practical thematic analysis as described by Saunders *et al*^20^ and based on the work of Braun and Clarke^21^ (reading and familiarisation; coding; and theming) as our qualitative research method. Data were coded using an open-source qualitative data analysis tool ‘Taguette’ .^22^

The research team comprised the authors. AE collected the data, assisted by JA and OF. All authors were familiarised with the data. AE and ER created the descriptive codes, and JA and OF reviewed these. AE and ER then used these codes (the codebook) to code the data. As suggested by Saunders *et al,*^20^ we deliberately used multiple coders with different perspectives of training, rather than a single coder (AE had recently finished IMT, and ER is a consultant and training programme director, for example) to mitigate the impact of any one coder’s perspective. AE and ER produced draft themes, which were sent to each other and JA and OF for reflections. The authors then discussed these and agreed on a common set of themes.

### Participant recruitment

We produced a voluntary anonymous survey which was sent to all stage 1 IMTs (Fig. 2) in London via the NHS England Training office, and by postgraduate medical education departments. It was also advertised on an *ad hoc* basis by the authors.

Respondents were asked if they would be willing to be contacted further, with those who responded ‘yes’ given the option of leaving their email address. These people were then contacted by email and invited to give their availability to attend a focus group and/or interview either in person or online. The authors then selected dates based on these responses to maximise the number of trainees who could attend through a mixture of online and in-person events. At the start of each session, participants were informed that recording was taking place, but that no identifiable information would be used in the final report. It was explained that the results of the sessions were being used for research as well as service development. Verbal consent was gained, and participants were reminded that they can withdraw their consent at any time including after the session, and that if they wished for certain comments or conversations to be positively excluded from the report then this would be respected.

### Data gathering and interview/focus group structure

The survey was produced on Microsoft Forms. All responses remained anonymous to the authors. The focus groups and interviews were conducted in a semi-structured manner and hosted by the authors either in person or online using Microsoft Teams. Sessions were transcribed using Microsoft Teams, and in-person events were also transcribed using OtterAI.^23^ Several prompt questions were agreed upon beforehand to stimulate discussion (appendix A) .

## Results and discussion

### Respondents, demographics, and theme generation

**Fig. 3:**
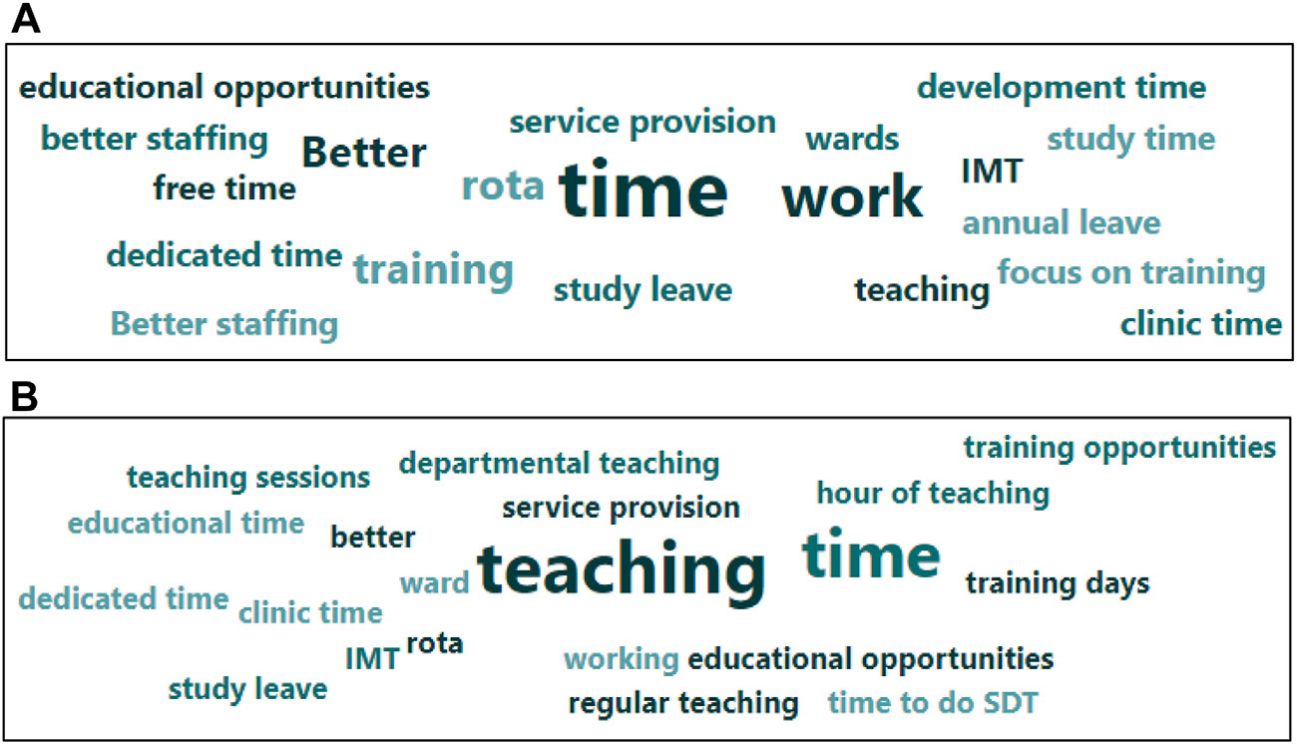
a. If I could do one thing to improve my wellbeing as an IMT, it would be: b. If I could do one thing to improve my education experience as an IMT, it would be:

156 IMTs responded to the initial survey. Demographic data are presented in Table 1. Answers to the Likert-type questions are presented in Fig. 2, with each question represented on a scale from ‘Strongly disagree’ to ‘Strongly agree’. Free-text responses provided insight into recurring and high-impact themes, and are presented in word-cloud form in Figs. 3a–b.

**Table 1:** Survey respondent demographics.

	IMTs (n = 156)	
Year of training	IMT1	27% (42)
	IMT2	44% (69)
	IMT3	23% (36)
	Prefer not to say	6% (9)
Age	<25	0% (0)
	25–34	89% (139)
	35–44	5% (8)
	>44	0% (0)
	Prefer not to say	6% (9)
Gender	Male	38% (59)
	Female	56% (87)
	Non-binary	1% (1)
	Other	0% (0)
	Prefer not to say	6% (9)
Ethnic group	White - English / Welsh / Scottish / Northern Irish / British	38% (60)
	White - Irish	3% (4)
	White - Gypsy or Irish Travelle	0% (0)
	Any other white background	10% (15)
	Mixed / Multiple ethnic groups - White and Black Caribbean	0% (0)
	Mixed / Multiple ethnic groups - White and Black African	0% (0)
	White and Asian	1% (2)
	Any other Mixed / Multiple ethnic background	1% (1)
	Asian / Asian British - Indian	14% (22)
	Asian / Asian British - Pakistani	4% (6)
	Asian / Asian British - Bangladeshi	1% (1)
	Asian / Asian British - Chinese	7% (11)
	Any other Asian background	4% (6)
	Black / African / Caribbean / Black British - African	1% (1)
	Black / African / Caribbean / Black British - Caribbean	0% (0)
	Any other Black / African / Caribbean / Black British background	0% (0)
	Arab / Arab British	1% (2)
	Any other ethnic group	2% (3)
	Prefer not to say	14% (22)
Full time vs less than full time	Full time	85% (133)
	Less than full time	11% (17)
	Prefer not to say	4% (6)
Place of primary medical qualification	UK	88% (137)
	Overseas	8% (13)
	Prefer not to say	4% (6)

Following the survey, 61 people said they were happy to be contacted further. Seven dates were arranged for interviews and focus groups, with four interviews and three focus groups conducted using the structure described above. In total, 15 trainees participated in these sessions (Appendix B, Table B.1) .

Through reading and coding of the transcripts, 27 codes were generated. These were further grouped into seven overarching themes: workplace culture; helplessness and distrust; individual circumstances; physiological needs/safety; rotas and work patterns; stress at work; and value (Table 2) .

**Table 2:** Themes, codes and code frequency, and example quotes.

Theme	Code	Number of codes	Example quote
Workplace culture	Bullying is reported as a cause for poor wellbeing	11	*Being free from bullying, from demeaning behaviour, insulting behaviour.*
	Culture in the workplace affects wellbeing	24	*I think the big thing [affecting wellbeing] would probably be the culture.*
	Individuals and/or personalities can positively or negatively impact an individual's wellbeing	19	*I think a lot of that [what improves wellbeing] is just the personality of the consultants.*
	Psychological safety inferred as an impacting factor	6	*I've been like with like consultants that I get on with and I know like, oh, can I ask a dumb question.*
	Teams that people work in, rather than the individuals within those teams, affect wellbeing	10	*Maybe you've got a [WhatsApp] chat. You know The Reg. You text each other. You get breakfast and it's really nice. But then sometimes you're like, yeah, different team every night.*
Helplessness and distrust	Helplessness is cited by trainees when discussing wellbeing, or methods to improve it	14	*It's not something you can you do this and all be fixed, it's just it's so large [the wellbeing problem].*
	Perfunctory approach to wellbeing from individuals and/or organisations	38	*Many of the wellbeing programmes don't understand how we work or what we do.*
Individual circumstances	External stressors outside of the workplace influence wellbeing	4	*My mental health [affects wellbeing].*
	International medical graduates often struggle with accessing wellbeing resources	2	*By their own admission, they [the GMC] said that they're profoundly racist organisation. I'm probably not going to be in that much trouble because I have a generically Western European sounding name.*
	Mental health used to define wellbeing as well as impacting it	5	*[Wellbeing is] how good you feel mentally and physically.*
Physiological needs/safety	Facilities such as rest facilities, availability of food and drink etc	7	*Suitable adequate rest areas with the acknowledgement that they will be used for rest.*
	Physical safety at work and home	3	*I would assume everyone's physically safe.*
Rotas and work patterns	Flexible working cited as a positive factor for wellbeing	25	*There's a lot of slack in the rota.*
	Less than full time working	2	*Going less than full time [would improve my wellbeing].*
	Rotas cited as an issue that influences wellbeing	44	*I think there's a lot to be done or could be thought about in terms of rota patterns and the coordination of the rota patterns to try and improve it [wellbeing].*
Stress at work	Burnout is reported as synonymous with wellbeing, or as a cause for low levels of wellbeing	21	*I was quite burnt out at the end of IMT2.*
	COVID-19 has or had an impact on trainee wellbeing	2	*Part of that [the drop in wellbeing] was due to COVID.*
	Staffing levels	14	*There is a persistent vacant rota line for every single rotation.*
	Stress at work is cited either as a definition of what wellbeing is or as something that influences it amongst individuals and/or teams	20	*It's the job, how stressful the job is.*
	Workload felt to be unmanageable	4	*I think part of it is the stress, because we are really busy… we are under intense pressure to discharge [patients] etc.*
Value	Education is cited as an influencing factor on an individual's wellbeing, either positively or negatively	44	*How well you're meeting your educational and learning objectives [affects wellbeing at work].*
	Feedback received either from peers or seniors	4	*When I was an F2, we had a consultant that was responsible for foundation doctors who arranged fortnightly meetings in order to help us with any issues that we were having.*
	Job satisfaction and wellbeing are intrinsically linked	10	*I enjoy being a doctor. IMT has such a bad reputation, but I'm not sure it deserves it.*
	Pay influences people's perception of their and their colleagues wellbeing	5	*Better pay [would improve wellbeing].*
	Supervisors, either informal or formal (educational, clinical etc) are mentioned as influences on trainee wellbeing	16	*I think really what [improved my wellbeing] was the frequency with which I could troubleshoot and meet with my supervisors.*
	Support at work, where the subject articulates feeling supported at work as contributing to their wellbeing, or lack thereof	34	*Consultant supervisors have always been quite supportive.*
	Value of one's self or one's role by others	33	*I think there's certainly something to be said about feeling valued at work.*

### Workplace culture

The culture of a workplace was a consistent theme across all sessions when discussing the causes of positive and negative wellbeing. On the extreme end of this, participants described episodes of bullying. For example, IMT-1 explained a scenario where they felt they had behaved safely and professionally in a situation where a number of staff were off sick on the acute medical take, only to be chastised for it the following day:

*There was only me and a Reg because the other two SHOs were sick and it was like I don't know how to manage these and I admitted everybody, ‘cause I was like, they all sound reasonable. And then I basically got shouted out for an hour on the post-take ward round the next morning for admitting everyone.*

Examples of this more overt bullying were not reported to be common, and participants commented more on a sense of a poor atmosphere. In one focus group, IMT-2 commented on how these situations can go unnoticed despite having a major impact on wellbeing:

*It's difficult to pick on specific examples because often it isn't about specific incidents or examples or saying that this particular person is a bully … it's really difficult to pin that down and therefore very, very difficult to address it and make changes about it because you can't put that feeling into writing or into a complaint.*

Individuals can have an equally if not more positive impact on wellbeing as well as a negative one, and the sessions were littered with examples of praise for consultants and supervisors and the difference they can make, for example:

*I think who I'm working with and like the support I've got has made a big difference -* (IMT-7)

Several participants, including IMT-7 and IMT-8, highlighted the fact that the culture in a team or organisation can be self-fulfilling, such that when poor culture is embedded it spreads to:

*[…] almost all levels because it starts trickling down*.

and that if seniors are seen to behave in a negative way, then it:

*[…] gives a licence that this is an acceptable way to speak to your juniors*.

### Helplessness and distrust

Respondents were often sceptical in their responses. IMT-1 doubted that their employers cared about their wellbeing enough to make a difference:

*If you're saying to people we acknowledge you're unhappy, but we're not going to do anything about why you're unhappy, we're only going to help you cope with being unhappy that screams of an employer and an organisation and a hierarchy that does not care.*

These respondents recognised that many organisations have some form of wellbeing provision but felt these were often at inaccessible or focused on the wrong things, or simply a ‘tick-box’ exercise. An example IMT-9 gave was:

*[…] these yoga things that were started at one of my trusts for lunchtime, no one clinical had time for that, but then they just sort of ticked the box and got and thought they'd done it.*

IMT-1 again summed up their distrust of the motives of organisations that push poorly designed wellbeing initiatives when they get bad feedback:

*And then some dude in a suit will go. ‘Hmm. Wellbeing numbers aren't so good. This is this is not looking good in the press’ and they'll be like ‘we shall wellbeing the crap out of them’.*

Many participants wondered whether any fixes were possible. IMT-2 spoke of the systemic issues and how these make fixing staff burnout and poor wellbeing levels so difficult:

*So much of the issues and the stresses were under also systemic … it's not something you can you do this and all be fixed, it's just it's so large.*

Despite this, a large number of trainees expressed how simply being listened to and being asked what they want or need has the potential to make a positive difference because at the moment:

*[…] many of the wellbeing programmes don't understand how we work or what we do -* (IMT-10) .

They had sympathy with those tasked with trying to improve the wellbeing of staff:

*[…] a lot of whom have not been a trainee for many, many years - (*IMT-11) .

Although helplessness and distrust were a recurring theme, in no session did this seem entirely irretrievable. As IMT-1 summarised, when designing and implementing wellbeing interventions:

*Give me something concrete.*

### Individual circumstances

External factors such as mental health conditions had a significant impact on some IMTs’ wellbeing. Feeling well:

*[…] mentally, physically, spiritually - (IMT-3)*

and

*[…] how good you feel mentally and physically -* (IMT-8)

as well as wider financial concerns volunteered by some were reported as determinants of their wellbeing.

International medical graduates also highlighted additional challenges affecting their wellbeing. IMT-14 observed that:

*International doctors are given very little autonomy, very little control, and they're told well, this is what you have to do to get in the system.*

### Physiological needs/safety

The BMA in 2018 produced a report entitled ‘Supporting health and wellbeing at work’,^24^ which concluded that environmental factors (eg rest facilities, changing areas), among other things, impact wellbeing significantly. Some have argued that there are in fact a hierarchy of factors, much like Maslow’s hierarchy of needs^25^^,^ such that attempting to improve *‘less important’* determinants of wellbeing will derive no benefit to the individual if their core needs, the more ‘essential’ determinants, are not already met.^26^ The hierarchy argument assumes that all people respond in the same way to interventions, and that wellbeing is the same for every individual. However, we would argue that for some people, ‘extra’ things at the top of the hierarchy can still improve their experience, and so should not be entirely ignored. Physiological needs and perceptions of personal safety would be the foundation of this pyramid, and these came up repeatedly as factors affecting IMT wellbeing. For example, while nobody reported feeling physically unsafe at any time, it was recognised that the risk of feeling or being unsafe was real and important. IMT-4 commented that:

*I think physical security working long shifts in A&E is definitely needed.*

The need for basic facilities were emphasised on multiple occasions. IMT-1 felt that:

*Providing hot food 24 h is incredibly important, [and] a place to sit down and document in any of the wards.*

IMT-7 felt that a:

*[…] little break room […] that stocks tea and coffee [without having to] sneak it out.*

would make a big difference to their wellbeing.

### Rotas and work patterns

Rotas, flexible working, and working patterns including less than full time working were frequently cited as impacting wellbeing, and the topic came up in every discussion with all participants. IMT-15:

*… felt most drained physically and mentally […] when you've been on a more chaotic or understaffed [rota].*

However, they felt more able to accept busy rotas if they could plan in terms of:

*[…] getting leave and being able to plan your life.*

IMT-5 succinctly made the point that

*I think a lot of the wellbeing side of it comes from the flexibility.*

Participants had a lot of respect for the complex work rota coordinators are often tasked with, and felt that they need the resources to be able to manage a rota effectively. As IMT-2 said:

*Being a rota coordinator is a very stressful job for people who often don't have a lot of information about our exact requirements. [We need to] support rota coordinators to be able to meet our needs.*

Trainees recognised that changes may be required, and that rotas must provide a service as well as cater to the wellbeing needs of staff. Indeed, many were also content with the fact that shifts may fall on important dates, but were frustrated when the system would not let them plan for this. As IMT-15 said:

*The fact that you're missing a weekend that you wanted to go to someone's wedding, that's gonna pull down your wellbeing. But if you feel cheated out of it because some part of the system didn't quite work and therefore you missed it, that's that makes you feel a lot worse.*

It is that very system that can turn a negative experience for wellbeing into a positive one, as IMT-9 explained with regards to a trust that do this very well:

*Here they try and give you your rota for the whole year and that's had a massive beneficial impact.*

### Stress at work

*People don't like to admit they're struggling - (*IMT-14) .

A number of the staff we spoke too had suffered from burnout, with some having taken time away from work to deal with this before returning.^15^ As is often the case, it is these very staff who struggle to recognise the signs in themselves, often going as far as to blame themselves for the very issues they are dealing with, as IMT-1 articulated:

*And I'm like, is this me? Am I the problem here?*

Even in trainees who would not identify as being burnt out, there are fears expressed when shifts are understaffed and frustration when this is not resolved. IMT-11 complained of a:

[…] *persistent vacant rota line.*

which they raised with medical staffing who they felt that:

*[they] reply to your emails like they're telling off a school child.*

This tone of response after raising concerns about staffing levels leads to a breakdown of trust in these and other reporting systems.

As with rotas being stretched, there was an understanding that working in healthcare is inherently busy, particularly in the current climate. As IMT-12 said:

*All of us are professionals. All of us can deal with busy.*

This did not necessarily mean that wellbeing would suffer. Indeed, some people:

*[…] actually, really enjoy it [the busy environment] - (*IMT-2) .

Being busy, however, is different to being burnt out. Burnout can often spread through whole teams or departments as described by IMT-15:

*When people are really drained, everyone's drained …[…] that's when the wellbeing of the whole place gets affected.*

Understaffing and overstretched departments can result in people not taking time to look after themselves, take breaks, or see to basic physiological necessities as discussed above like having a meal. When asked what could be done to improve this, the response from IMT-6 was simple:

*Ensure the ward is well staffed enough so that you can have a decent lunch break.*

Several trainees linked the stress they experience at work with the pressure that they feel under to keep their training and portfolios up to date, for example IMT-9, who said that:

*I link it quite a lot with burnout and how much capacity we've got for keeping our portfolio up to date.*

IMT-11 described how there is a mismatch between promised study leave and the reality of having to stay on the wards and consequently miss:

*[…] every single regional teaching day this year […] I need time to catch up on these sessions that are 4 h long and then there's no time for you to take study leave so we spend our zero days, you know, catching up on teaching that's supposedly mandatory.*

### Value

Much of what we have discussed above can be summarised in one word: value. For some including IMT-8, value meant literal value in terms of:

*Better pay.*

There was a sense that years of pay constraint particularly during and following the sacrifices made during the COVID-19 pandemic was evidence of not being valued in the NHS broadly.

The importance of valuing training and education was also a consistent theme. We heard several excellent examples of when individuals and departments made valuing a trainee’s education a priority. For example, simply:

*Having clinics scheduled into your rota.*

to help with mandatory clinic requirements as IMT-6 had. Some were able to go even further, for example in IMT-3’s trust, where:

*Consultants have a ticket clinic where you could go to them for a certain hour each day to do CBDs (case-based discussions) and Mini-CEXs (mini clinical evaluation exercises) .*

Unfortunately, there were as many examples of where trainees felt that their training was not considered a priority, such as when:

*One of our IMT trainees had to make five swaps just to get her Part 1 [MRCP examination]* - (IMT-10) .

Having to pay for these either because study budgets do not cover them (eg exams) or because the systems in place to claim expenses felt opaque (eg courses and conferences) very much had the effect of demonstrating in pounds and pence how little value was placed on education. As IMT-1 observed:

*We shouldn't be one of the only industries that has to pay for their own exams, pay for their own training courses, pay for their own work materials.*

Feeling valued also meant that trainees were more engaged and felt part of a team. IMT-1 explained the difference they felt moving from an unsupportive environment to one where they felt valued, and their wellbeing was looked after:

*Now I feel like I could make changes in the department. I could suggest changes and I already have, and I've seen them be acted on and they've made work better.*

The importance of being listened to and seeing one’s suggestions being acted on cannot be understated. Seniors asking for feedback and listening to concerns where:

*[…] a genuine effort was made to try and rectify those issues.*

as described by IMT-6 demonstrated to trainees that their feedback was not only gathered, but heard and acted upon.

Support often came from:

*[…] very supportive, very accessible educational supervisors who are […] present should you want to have a conversation, who are accessible should there be any problems - (*IMT-11) .

Overall, trainees felt:

*On the whole that we have very accessible local TPDs (training programme directors)* - (IMT-13) .

Of course, the converse was also true, and IMT-14 put their negative experience down to one key aspect:

*I lost trust in my supervisors.*

Trainees were all understanding that supervisors as well were under pressure and at risk of burnout, often struggling with the same issues that they were, so were even more grateful when they felt a supervisor value them.

## Discussion

### Limitations

There are several limitations to our research. As both the survey and follow-up qualitative research was voluntary, participants are self-selecting and unlikely to be fully representative of all IMTs. Indeed, GMC data show that trainees who are at greatest risk of burnout are the least likely to know who to contact if they needed support and so are less likely to be engaged with this type of study, despite being the very group to whom interventions should be targeted. Furthermore, while we have attempted to contact IMTs via multiple routes of communication, we cannot be sure that all IMTs in the region received our invitation to participate, which may further distort the sample population.

Our sample population demographics will not exactly match that of the overall IMT population. For example, participants in our research were disproportionately in the second, ie IMT2, year of their training (Table 1), which may skew the responses to issues that affect them more than those at the beginning or end of their IMT journey.

Researcher bias exists in qualitative research, and we have adopted a strategy of openly acknowledging these biases and attempting to embed reflexivity proactively into our work at all stages through from study design to the framing of this paper.^27^

### Recommendations

We recommend several practical interventions for organisations to implement to improve the wellbeing of their employees, specifically their trainees, to urgently address the themes we have identified.

1. *Workplace culture.* Organisations should build a culture of personal and psychological safety where bullying and intimidation are not tolerated. This comes from the top, with leaders demonstrating responsible behaviours. Signing up to and enacting the NHS People Promise is a minimum requirement.^28^ It also requires having processes in place to report and deal with situations when high standards are not met. Training for educators and supervisors should be provided to help build this culture. Active bystander training should be utilised to empower staff to speak up and challenge poor behaviours and support victims.
2. *Helplessness and distrust.* Trainees must see and believe that their wellbeing is a priority and that their concerns will be addressed. All trusts are required to have a Freedom To Speak Up Guardian and Junior Doctor Forums; these must be well advertised at both regional and local inductions and require buy in from senior leaders for legitimacy. We suggest that, furthermore, organisations should identify a senior named individual responsible for the wellbeing of trainees. Trainees should have input into who takes up these roles to ensure that they carry the trust of those they advocate for. Changes and improvements should be fed back to trainees when they occur. NHS England should commit to the ongoing funding of wellbeing and education fellows^8^ following successful pilots over the last academic year.
3. *Individual circumstances.* Everyone involved in postgraduate medical training should be flexible with regards to individual circumstances. This applies to NHS England and NHS trusts, as well as to TPDs and trainee supervisors. Some of these circumstances will be predictable, for example, maternity leave and supported return to work – resources should be readily available for this. An example of good practice is the Wellbeing Directory for Doctors in London, which details the national, regional, and trust- and site-specific resources available at every hospital in London, and we would advocate each region or deanery having similar information available.
4. *Physiological needs/safety.* Trainees have a right to feel safe at work and have access to basic amenities. Rest facilities should be free and easily accessible, hot food should be available 24/7, and these should be openly and repeatedly advertised to staff. Every organisation must adhere to the BMA Fatigue and Facilities Charter as a minimum requirement.^29^
5. *Rotas and work patterns.* NHS England have recently restated that work schedules should be received 8 weeks in advance and finalised duty rosters 6 weeks in advance, and have committed to providing information regarding incoming doctors to organisations within the requires 12-week time frame to facilitate this.^30^ This is a minimum and yet essential requirement and cannot be overstated. We would go further and suggest that organisations commit to providing rotas as far in advance as possible and for as long as possible, with the option of self-rostering where possible. Medical staffing and rota teams should be adequately resourced to achieve this, and examples of good practice should be shared locally and regionally.
6. *Stress at work.* Organisations and individuals should take steps to identify those at risk of burnout though national and local surveys. Supervisors should receive education in how to identify these trainees, including through accredited continuing professional development as well as the development of strong local networks of educators from supervisors to TPDs. Strategies to improve this should be designed with the input of trainees, who must also remain aware that their seniors and supervisors are at risk of burnout as well as them.
7. *Value.* Finally, and most importantly, trainees must be valued. Self-development time must be rolled out for all trainees, as it already is for foundation trainees and some higher specialty training programmes. This should be built into duty rosters. Study leave for exams should be granted prospectively. Funding for mandatory courses should be paid in advance, rather than having to be claimed back retrospectively. The government must recognise the impact of pay erosion and commit to reversing this. Organisations and government should recognise the financial burden of training and take steps to address this.

## Conclusion

Our work surveyed over 150 IMTs in London, followed by targeted focus groups and interviews to gain further qualitative insight into the issues faced. After transcript reading and familiarisation and coding, we identified seven key themes related to wellbeing: culture, helplessness and distrust, individual circumstances, physiological needs/safety, rotas and work patterns, stress at work, and value.

## Funding statement

This research did not receive any specific grant from funding agencies in the public, commercial, or not-for-profit sectors.

## Data availability statement

The data that support the findings of this study are available from the corresponding author upon reasonable request.

## CRediT authorship contribution statement

**Alexander Emery:** Writing – review & editing, Writing – original draft, Methodology, Investigation, Formal analysis, Data curation, Conceptualization. **Jesal Acharya:** Writing – review & editing, Investigation. **Oliver Fox:** Writing – review & editing, Data curation. **Emma Rowlandson:** Writing – review & editing, Supervision, Conceptualization.

## Declaration of competing interest

The authors declare that they have no known competing financial interests or personal relationships that could have appeared to influence the work reported in this paper.

## Figures and Tables

**Table B.1:** Distribution of respondents.

Interview / focus group	Anonymous respondent code
Interview 1	IMT-1
Focus group 1	IMT-2
	IMT-3
	IMT-4
	IMT-5
	IMT-6
Focus group 2	IMT-7
	IMT-8
Interview 2	IMT-9
Focus group 3	IMT-10
	IMT-11
	IMT-12
	IMT-13
Interview 3	IMT-14
Interview 4	IMT-15

## Appendix A

- Introductions
- What is wellbeing, and what does wellbeing mean to you?
- What do you think wellbeing means to NHS trusts or your employers? Do you think the definition you have is different?
- Think of one job or rotation when you think your wellbeing was well looked after. What was is about this that made you feel that way?
- Think of one job or rotation when you think your wellbeing was poorly looked after. What was it about this that made you feel that way?
- If I gave you £20 million to improve the wellbeing of medical trainees, what would you do?
- If you had limited resources but were asked to improve the wellbeing of medical trainees, what would you do?
- Any other comments
